# Influence of Fitness Apps on Sports Habits, Satisfaction, and Intentions to Stay in Fitness Center Users: An Experimental Study

**DOI:** 10.3390/ijerph181910393

**Published:** 2021-10-02

**Authors:** Manel Valcarce-Torrente, Vicente Javaloyes, Leonor Gallardo, Jerónimo García-Fernández, Antoni Planas-Anzano

**Affiliations:** 1 Department of Business Management, Universidad Internacional de Valencia (VIU), 46002 Valencia, Spain; 2 Centro Lleida, Department of Sport Management, Instituto Nacional de Educación Física de Cataluña, 08038 Barcelona, Spain; vjavaloyes@inefc.es (V.J.); aplanas@gencat.cat (A.P.-A.); 3 IGOID Research Group, Physical Activity and Sport Sciences Department, Universidad de Castilla-La Mancha, 45004 Toledo, Spain; leonor.gallardo@uclm.es; 4 Research Group of Management and Innovation in Sports Science, Leisure and Recreation (GISDOR), Universidad de Sevilla, 41013 Seville, Spain; jeronimo@us.es

**Keywords:** fitness app, satisfaction, loyalty, fitness industry, experimental study

## Abstract

The use of technology in sports and fitness is proliferating thanks to advances to facilitate its practice and improve adherence. Beyond adherence, it is important that technology is understood as a facilitating medium. The main objective of this study is to know the influence of the use of the fitness application (app) on sports habits, customer satisfaction and maintenance intention of fitness center users. For this, an experimental, controlled and randomized study was carried out, characterized by being a field trial, with a sample of 66 participants divided into a control group (*n* = 33) and an experimental group (*n* = 33), with 38 (57.6%) men and 28 (42.4%) women who self-monitored their physical activity for 8 weeks. The dimensions analyzed between the pre- and post-intervention phases were the changes in their sporting habits (frequency of attendance and duration of the session), the changes in satisfaction and the intention to stay with respect to the fitness center. The results in general do not show significant differences between the two groups and conclude that the use of the fitness app did not directly influence the sports habits of the participants. There were also no significant differences in terms of satisfaction with the fitness center or in their intention to stay in the fitness center. Therefore, it is shown that the use of the fitness app, as a single download or use element, is not enough to improve habits, satisfaction or the intention to stay in the fitness center.

## 1. Introduction

From the beginning of the fourth industrial revolution several years ago, digitalization and the use of the internet in societies continues growing. It is worth noting internet access through mobile phones reached 5.22 billion devices in January 2021, assuming a penetration of 67% worldwide [1]. In the world, 55.7% of total web traffic is made from mobile phones, dedicating 91% of the time we use to mobile phones for the use of applications, spending an average of 4.2 h per day per person. In 2020, a total of 218 billion applications (apps) were downloaded in the world, with a total expenditure of 134 billion dollars, 20% more than in 2019 [2].

Sport, like digitization, continues growing and evolving towards a greater organization and professionalization in their strategies, resources, staff and business processes [3]. In Spain, it generates a total of 39,117 million euros, 3.3% of the national GDP, and assuming the creation of 414,000 employees, 2.1% of the total employed population, confirms the great economic and social impact for society [4]. The fitness sector, for its part, is currently considered a global commercial phenomenon [5], which has been evolving and diversifying its business models and the offer of its services, orienting them to the improvement of people’s health through the performance of qualified professionals [6]. Currently, the fitness sector has a turnover of 96.7 billion dollars worldwide, with a total of 184 million users spread over 210,000 fitness centers [7]. Precisely in Spain, 2352 million euros were invoiced in 2019, accumulating a total of 5.5 million users in its 4743 fitness centers [8].

Faced with the current pandemic situation produced by COVID-19, fitness centers have the challenge of continuing to apply and implement digitization to continue growing and adapting to the new formulas for user relationships and consumption. The changes produced by digitalization also affect the administration and management of sports facilities, as well as the provision of services and their relationship with customers. [9]. A growth in the use of apps related to health and fitness has also been observed in order to promote physical exercise and knowledge of the condition and health by users [10]. For this reason, an increase in the number of apps developed related to health and fitness has been observed in recent years, just as the interest in their scientific study [11].

Based on the aforementioned information, the aim of this study is to know the influence of fitness apps on sports habits, satisfaction and the intention to stay in fitness centers. The study is structured in a second section that includes the theoretical foundation that explains how the use of technology affects the behavior of users in the cited variables, and a third section that develops the applied methodology and argues the procedure carried out. The main results are developed in the fourth section, structured according to the main three variables analyzed. The study is concluded with the discussion of the findings and proposing the main applications of the study and its limitations and future lines of research.

## 2. Theoretical Foundation

### 2.1. Technology and Sports Habits

Estrada-Marcén et al. observed that more than 60% of fitness center users used technology to perform their workouts [12]. It is true that they were not the fitness center’s own app and that, above all, they were used for running or indoor cycling activities. This study also concluded that 72% of users considered that the use of exercise apps motivated them towards the achievement of their daily physical practice goals. In this sense, there is early evidence about apps that improve healthy lifestyle habits are well received by users [13].

However, there is no clear consensus in the literature about the influence of the use of fitness apps on the sports habits of users directly. In some cases, no significant differences were found [14,15,16], while in others, they perceived a correlation between the use of apps and the frequency of training, the duration of training program and the number of participants [17,18]. The most recent studies indicate the importance of extrinsic factors that intervene in the use of apps to improve sports habits such as the environment, the type of training, accessibility, interaction or motivation [19,20,21,22].

Based on the above, the first hypothesis proposed is:

**Hypothesis** **1 (H1).** *Technology in fitness centers influences the sports habits of its users.*

### 2.2. Technology and Customer Satisfaction

Beyond the use of the technological device, it is important that it be focused on the practice of physical exercise itself, and even on the loyalty of the facility or entity that provides physical exercise services. That is, technology understood as a facilitating way. In fact, technology could be part of the customer’s perceived value, directly related to perceived quality and, therefore, also to satisfaction [23].

In a recent study by Gelbrich et al. (2021) based on four interventions, it was concluded that the use of technology based on emotional support and follow-up through a virtual assistant increased customer satisfaction and persistence due to higher perceived satisfaction [24]. Therefore, they consider that digital services not only offer information, guidance and suggestions, but their opportunities also increase through technology and artificial intelligence, and they can also offer emotional support, expressing empathic and reassuring expressions with customers in achieving your goals.

Based on the above, the second hypothesis proposed is:

**Hypothesis** **2 (H2).** *Technology in fitness centers influences the customer satisfaction of the fitness users.*

### 2.3. Technology and Maintenance Intention

The widespread use of mobile phones and apps has meant that fitness centers increasingly implement their own apps with the aim of generating greater adherence to physical exercise and loyalty to the fitness center itself [25]. This is due to the fact that the use of apps and other digital platforms provide more segmented information on customers, encouraging the personalization of services to their stakeholders and, therefore, developing strategies adapted to their needs [26].

On the other hand, it should stand out that the degree of permanence and loyalty is related to the commitment and continued use, in this case of mobile applications, as indicated by the studies by Carlson and O’Cass [27] and Flurry Analytics [28]. The latter concluded by tracking more than a million applications that users who used the fitness and health applications three times a week obtained retention rates above 40%. It also found that more than 75% of users opened their application related to fitness and health at least twice a week and more than 25% accessed the application more than 10 times a week, being able to consider them as “addicts”, highlighting the importance of continuous and constant use to achieve the proposed objectives. More recently, the study by Chiu et al. (2020), who analyzed the intention to continue using apps, concluded that greater use, frequency and commitment to the application could help to improve the retention and loyalty rates of fitness center users [10].

Based on the above, the third hypothesis proposed is:

**Hypothesis** **3 (H3).** *Technology in fitness centers influences the intention to stay of fitness users.*

## 3. Materials and Methods

### 3.1. Study Design

An experimental study was carried out where the sample was integrated into two groups, a control group (CG) and another experimental group (EG). This type of experimental design was chosen for the advantages that it offers in relation to the strength of the causality inference, giving answers to the causes on specific phenomena [29]. Both groups were given an initial and a final questionnaire. The difference was in the type of intervention they received: support with the fitness app (EG) or traditional support without the use of the fitness app (CG).

### 3.2. Participants

Eligible participants were those who, being volunteers, fulfilled the inclusion criteria of: (i) between 18 and 54 years of age, (ii) had less than three months’ seniority as a customer fitness center and (iii) who had a smartphone; and the exclusion criteria consisting of: (i) genders other than male or female, (ii) people with health disorders that could cause loss or alter the results of adherence due to health situations that could generate them, such as macrovascular damage, deficiency diseases, mental disorders, toxic addiction or sensory deprivation, (iii) people with previous experience with exercise applications linked to the fitness center and (iv) not having previously been a customer of the fitness center.

For the selection of the sample, the study was carried out at the Universidad Europea Sports Complex, on the Villaviciosa de Odón Campus in Madrid, which has a fitness center. The recruitment of participants was carried out in collaboration with the management, reception staff and sports monitors through their database. Subsequently, a telephone call was made to all of the users who met the participation requirements to offer them the possibility of participating in the study and to summon them to sign the informed consent, fill in the initial questionnaire and provide information on the assignment to a group. If they were willing to be study subjects voluntarily, and after summoning them, they proceeded to sign the informed consent and fill in the initial questionnaire.

Once the participants were received, four of them were selected, and after analyzing the results of the initial questionnaire in relation to the inclusion and exclusion criteria, they were randomized into the corresponding CG and EG. All of the target participants who were volunteers received an email indicating whether or not they had been selected. Those who were selected also received a call to explain the process in more detail, especially those who were randomized in the EG, information that they also had available by email.

The sample (Table 1) was made up of a total of 66 participants, with a mean age of 21.84 years in the CG and a mean age of 22.48 years in the EG. Regarding gender, we found that 51.5% (*n* = 17) of the CG participants were male and 48.5% (*n* = 16) female. On the part of the EG, 63.6% (*n* = 21) were male and 36.4% (*n* = 12) were female.

Concerning the level of studies of the participants, we highlight that in the CG, 75% (*n* = 24) were school graduates, and 72.7% (*n* = 24) were in the EG. The majority, as observed in both groups, more than 70%, (*n* = 48) coincide in having a school graduation.

### 3.3. Instruments

Different phases were used to prepare the questionnaire. First, an intensive review of the literature related to the variables to be analyzed was carried out. Subsequently, a group of experts met to obtain an instrument that would answer the research questions. In particular, the concepts of each variable to be analyzed in fitness centers were determined. The most relevant studies were reviewed in order to quantify the variables. Subsequently, the measures as well as the items to be used were determined. Finally, a consensus was reached on the instrument to be used, which is described below.

The instrument consisted of two questionnaires, one prior to the intervention and the other at the end of the eight weeks. In the first, variables that could be associated factors and confusing the results were also incorporated, as well as those in order to exclude those subjects who did not meet the predefined criteria.

The first part of the questionnaire was made up of the sociodemographic variables: gender, age and level of studies; and sports habits: weekly practice frequency (one day, between 1 and 2, between 3 and 4, 5 or more days), duration of the training session (from 30 to 60 min, from 60 to 90 min, more than 90 min), level of fitness experience (low level, medium level, high level) and type of activity carried out (individual sport, collective sport, fitness/bodybuilding, fitness/bodybuilding/sport, individual and collective sport). These variables have been used in other related studies [18,30,31].

A second part was related to the variable customer satisfaction with the fitness center, measured through the adaptation of CALIDFIT [32], since in addition to having been designed specifically for fitness centers, it was validated in Spain. This instrument is made up of five variables (the fitness center’s staff; the facilities and material; the services and activities; the quality/price ratio; and the general impression of the organization). For this, an item was adapted for each of the variables, taking as a response a Likert-type scale of 1 to 5 points (1 strongly disagreed and 5 strongly agreed).

Finally, a third part linked to the intentions to stay in the fitness center was included, one of the issues that most concerns its managers [33]. A questionnaire with seven items was carried out on what would be the intentions to change the fitness center, with a Likert-type scale from 1 to 5 (1 = nothing; 5 = a lot). To the question “what would motivate you to change your fitness center?”, the following response items fit: better price, closer to the workplace, closer to home, better equipment, better facilities, better technical equipment, better technological equipment.

### 3.4. Intervention

The intervention lasted eight weeks, during which the subjects self-monitored their sports activity, either in the EG or in the CG. In the first, it was carried out through the fitness app Fitbe (Valte Investment, Seville, Spain), in which their activity was recorded and routines were assigned by the trained sports monitor, and where the participant was also able to assign routines independently and even participate in gamification actions to achieve goals. In the CG, self-monitoring was carried out in the traditional way, with the assignment of manual routines (on paper).

The EG participants were previously informed about the use of the fitness app, receiving an email with their access data, username and password. They entered their personal profile data in it and began to experiment and use it. After accessing the fitness app, a training plan was assigned by the sports monitor.

For the correct use and adaptation to the fitness app by EG participants, a series of interactions were carried out through the app each of the eight weeks that the intervention process lasted (Table 2).

During the eight weeks, the CG followed the usual routine that is developed in the fitness center, which in this case consisted of the assignment of basic physical conditioning programs by the sports instructors to all of those participants who requested it, since it is a service from the fitness center itself. No type of self-registration of the activity and training was implemented, and no additional challenges were carried out with these participants, beyond the exceptional activities of the fitness center itself, to which those of the EG also had access.

### 3.5. Analysis of Data

To summarize the study variables, the descriptive statistics indices were calculated: for qualitative variables, frequencies and percentages were presented; for quantitative variables the mean and standard deviation were presented. Comparisons were made at the initial moment to verify that the groups were not out of balance.

To analyze the relationship between two qualitative variables 2, tests were applied, in 2 × 2 tables with the Yates correction, and to evaluate the effect size (effect size), Cramér’s V was applied [34]. For the comparison between independent samples, the application assumptions were checked: normality using the Shapiro–Wilk test, and homoscedasticity using the Levene’s test.

To compare groups, the Mann–Whitney *U* test was applied since the EG violated normality and the effect size was determined using the biserial correlation coefficient. To verify the effect of the intervention, the repeated measures ANOVA 2 × 2 split-plot test was applied, making exhaustive comparisons (post hoc) with the Tukey correction. Statistical analyzes were performed with the JASP version 0.13 program (Department of Psychological Methods, University of Amsterdam, Amsterdam, The Netherlands).

## 4. Results

### 4.1. Sports Habits

Regarding the sports habits of the participants in the initial moment prior to the intervention (Table 3), no statistically significant differences were observed between the groups in any of the variables analyzed, an issue that is positive in order to start with a homogeneous sample, as specified in the methodology.

It is noteworthy to highlight a more moderate effect between both groups in the variable weekly frequency (V = 0.300) and in the variable type of activity (V = 0.352), but without relevant significance.

Concerning the analysis of the sports habits between both groups once the intervention process had been carried out (Table 4), a non-significant increase was observed in the EG (M = 7.5; *p* = 0.058) but with a moderate effect (V = 0.349) in the weekly frequency of attendance at the fitness center since the number of participants who attend 5 or more days increases from four participants in the pre-intervention to seven in the post-intervention.

Regarding the variable duration of the session, in both groups, a similar increase in the time spent in the fitness center was observed, although it was not significant. In both cases likewise, its effects are moderate; V = 0.398 in the CG, and V = 0.319 in the EG.

### 4.2. Fitness Customer Satisfaction

Concerning the analysis of the changes in the appreciation of the customer satisfaction with the fitness center between both groups, once the intervention process has been carried out (Table 5), variable 2 (facilities and material) obtains statistically significant differences between the CG and EG with a mean effect between score differences. The CG (Mdif = −0.400) values the facilities and material with greater satisfaction than the EG (Mdif = −0.4).

On the other hand, customer satisfaction with the fitness center increases in all variables, with a mean effect of V1 (fitness center staff) and V2 (facilities and material), while V3 (services and activities) with F = 18,496, V4 (quality/price ratio) with F = 13,157 and V5 (impression of the organization) with F = 10,975. These changes have an important change between the before and after moment.

### 4.3. Intention to Stay in the Fitness Center

Finally, in reference to the evaluation of the changes in the intention to stay in the fitness centers of the participants of both groups (Table 6), variable 5 (best facilities) obtained statistically significant differences between the CG (Mdif = −0.645) and the EG (Mdif = −0.536) with a mean effect between the score differences. The EG (*p* = 0.231) has a greater intention to change fitness centers according to this variable than the CG (*p* = 0.082).

Regarding the variables v2 (closer to the workplace), v3 (closer to home), v5 (better facilities), v6 (better technical equipment) and v7 (better technological equipment), the scores on intention are increased about the intention of changing fitness centers, with such changes having a significant effect between the time before and after their analysis.

## 5. Discussion

The aim of this study was to know the influence of fitness apps on sports habits, customer satisfaction and the intention to stay in fitness centers, posing three hypotheses. On the first, related to sports habits, and in particular to the frequency of attendance, a determining factor for adherence to the fitness center, we did not find significant differences between the two groups, and most attend between three and four times a week, 57.6% (*n* = 19) for the CG and 51.5% (*n* = 17) for the EG. These results coincide with other studies carried out [14,16].

However, there is an increase in the frequency of attendance in the experimental group compared to the initial data, with attendance increasing by 19.2% between three and four times a week, while maintaining the control group. This aspect indicates a slight tendency to significance, only offset by a decrease in attendance five or more times a week. This tendency to significance is demonstrated by other studies by Kohler et al. and Smith and Biddle, who relate the increase in adherence with the frequency of training [18,30].

Related to the duration of the training sessions in the fitness center, we find the same difference of 15% greater between the control and experimental groups than at the beginning in the sessions of 60 and 90 min, but also an increase of 10% was observed in this range in both cases compared to the initial data, to the detriment of sessions of more than 90 min. There are therefore no significant differences between the two groups, and it seems unlikely in this case that the use of the application has affected the duration of the users’ training sessions, as in other studies [14,15,16], but it is observed that there may be greater efficiency in training and their duration, as in the studies by [17,18,30]. As stated by [14,34,35], it will be necessary to observe whether the use of the application consistently and maintained over time significantly influences the sports habits of users, comparing the results in different longitudinal studies that have larger time scales. As indicated by Ritterband et al. (2009), the use of technology helps to change behavior by improving both technical knowledge and experience towards achieving achievements, a fact that occurs when using the application in the visualization and development of training [36].

On the other hand, Barkley et al., Canhoto and Arp and Helander et al. comment on the importance of application use factors and how they can influence the user’s sports habits [29,30,37]. The fact of using the application for fitness and bodybuilding training activities would help to produce an increase in this type of practice in the participants. The establishment of personalized sessions and objectives linked to the type of training in the fitness room and bodybuilding could have led to a change in the trend in the types of activity carried out by the participants, as argued by Morgan et al., Picorelli et al. or Wang et al. [21,38,39].

Regarding the second hypothesis, and if there are changes in the evaluation of the customer satisfaction with the fitness center in both the control and experimental groups, we observed an increase in the same in variable 1 (fitness center staff), variable 2 (facilities and material), variable 3 (services and activities), variable 4 (quality/price ratio) and variable 5 (impression of the organization). In the case of V1 and V2, the effect of this increase was medium, while in V3, V4 and V5, its effect was significant.

Concerning variable 2 (facilities and material), statistically significant differences were obtained between the control and experimental groups with a medium effect. In this case, the control group valued the facilities and material with greater satisfaction than the experimental group. These increases in the perception of satisfaction by the participants coincide with the articles published by García-Fernández et al. [31,40].

Finally, the maintenance intention in the fitness center is one of the issues that most concern managers. It depends on the loyalty of users of fitness centers, either from a behavioral or attitudinal perspective, or integrating the previous two [32,41]. Knowing its degree of variation based on the analyzed variables will help us to appreciate the intentions of users to stay in the fitness center.

In relation to the control group, the results showed differences in most of the dimensions analyzed. The variables that changed significantly were v2 (closer to the workplace), v4 (equipment), v5 (better facilities) and v7 (better technological equipment). Regarding the experimental group, significant differences were also observed at a general level. The dimensions that varied most significantly were v2 (closer to the workplace) and v6 (technical equipment). The result of these data confirms those produced in other studies where the fact of encountering users with a high perception of the quality and satisfaction of the fitness center influences their degree of permanence in the fitness center in a positive way [42,43].

In relation to the third hypothesis, which postulates the influence of technology on the intention to stay in a fitness center by its users, we have not found related studies to date. In any case, the results show us both in the control group and in the experimental group significant positive results, being higher in the control group, so we cannot affirm that it is an exclusive element that affects the fidelity levels. However, it should be emphasized that loyalty is related to commitment, repeated practice or continued use, in this case of mobile applications and technology, as indicated by the studies by Carlson and O’Cass, Chiu et al. and Flurry Analytics [10,27,28], and therefore, greater use, frequency and engagement with the application could help improve the retention and loyalty rates of fitness center users.

### 5.1. Limitations and Future Lines of Research

The main limitations found are related to the recruitment capacity, both due to the difficulty in finding fitness centers willing to carry out a study of this type, as well as the target participants themselves, with a high volume who did not want to volunteer for the study.

Regarding future lines of research that give continuity and depth to this study, it would be considered interesting to be able to develop longitudinal studies, with wider temporal spaces, being able to compare between them and thus obtain results with a broader vision of the influence of the use of applications of the own fitness centers on their users.

Another of the lines of research this study could improve would be the analysis based on the client’s profile, taking into account age, gender or socio-demographic condition. In this way, we would know its influence according to user segmentation, considering the different generations and how they are linked to the use and acceptance of technology. In addition, it would be of great interest to be able to analyze the influence and use of mobile applications according to the business model of the fitness center, as well as the method of implementation of the mobile application of each fitness center, assessing its impact on users and their differences in function of the aforementioned business model.

### 5.2. Practical Implications

As for recommendations, a comprehensive implementation plan of the fitness center will be necessary, involving the staff, the user and their training plan, with adequate monitoring of the use of the application and a system of both individual and group challenges that keep the team committed and allow you to achieve your goals.

The digital transformation of sports facilities not only affects a first layer of management of processes, protocols or tools that facilitate their development, but also affects a second layer of consumption, practice, relationship and user experience. Success will lie in the continuous and long-term use by the user, becoming an element that helps their motivation, generating adherence, repetitive behavior and an attitude that generates satisfaction, and therefore loyalty.

## 6. Conclusions

Based on the findings, it can be concluded that the use of mobile phones applications from the fitness center itself by its users without previous experience did not directly influence their sports habits, their satisfaction or intention to permanence since there are no significant differences between the control and experimental groups between the pre- and post-intervention phases of the study.

With regard to if technology in fitness centers influences the sports habits of its users, no significant differences were found between both groups on the frequency of attendance; therefore, it is concluded that the use of the fitness app did not influence this variable. However, a slight trend towards significance was observed with a greater increase in gym attendance in the post-intervention phase. Regarding the duration of the training sessions, it is concluded that there were no significant differences between the control group and the experimental group between both study phases, but there was a trend towards significance in the experimental group, where the duration of training was increased.

With regard to if technology in fitness centers influences the customer satisfaction of the fitness users, in relation to customer satisfaction with the fitness center, no significant differences were found between the control group and the experimental group between both study phases.

With regard to if technology in fitness centers influences the intention to stay of fitness users, it is concluded that this hypothesis is rejected since there were no significant differences between the control and experimental groups both in the pre-intervention phase and in the post-intervention phase.

The conclusions therefore show that the use of technology and mobile applications, as the sole download or use element, are not enough to modify the sports habits, satisfaction or the intention to stay of the users of fitness centers.

## Figures and Tables

**Table 1:** Statistical data of the study population.

Variables		CG		EG	
		M	SD	M	SD
Age		21.84	2.2	22.48	3.8
		**N**	**%**	**N**	**%**
Gender	Male	17	51.5	21	63.6
	Female	16	48.5	12	36.4
Level of studies	Less than school graduate	0	0.0	2	6.1
	School graduate	24	75.0	24	72.7
	Formative cycle middle or higher grade	3	9.4	5	15.1
	Bachelor’s or university degree	4	12.5	2	6.1
	Postgraduate (master’s degree or doctorate)	1	3.1	-	-

M = mean; SD = standard deviation; CG = Control Group, EG = Experimental Group.

**Table 2:** Interactions with the experimental group in the intervention process.

	Interaction	Response	Consequence
Start	Do you have any questions about the use of the fitness app?	YES/NO	NO, Contact
Week 1	Have you done any more training in addition to the one assigned?	YES	Registration in app
Week 2	How many personal achievements have you managed to unlock?	YES-register	Registration in app
Week 3	Have you updated your weight?	Activation	Use of app
Week 4	Calorie challenge. Induced gamification.	Activation	Use of app
Week 5	How is using the fitness app helping you in your training?	Activation	Use of app
Week 6	Distance challenge. Induced gamification.	Activation	Use of app
Week 7	Are you fulfilling your plan of assistance to the fitness center?	Activation	Use of app
Week 8	Time challenge. Induced gamification.	Activation	Use of app

**Table 3:** Description of the sample at the beginning: sports habits.

	Control Group		Experimental Group				
**Weekly Frequency**	**fc**	**(%)**	**fc**	**(%)**	**χ^2^**	***p***	***V***
One day	1	3.13%	0	0.00%	5.682	0.128	0.300
Between 1 and 2	7	21.88%	14	45.16%			
Between 3 and 4	8	56.25%	10	32.26%			
5 or more days	6	18.75%	7	22.58%			
**Session Duration**	**fc**	**(%)**	**fc**	**(%)**	**χ^2^**	***p***	***V***
From 30 to 60 min	9	29.03%	4	13.79%	2.391	0.303	0.200
From 60 to 90 min	17	54.84%	21	72.41%			
More than 90 min	5	16.13%	4	13.79%			
**Fitness Experience Level**	**fc**	**(%)**	**fc**	**(%)**	**χ2**	***p***	***V***
Low level	6	20.00%	4	12.90%	0.882	0.643	0.120
Medium level	17	56.67%	21	67.74%			
High level	7	23.33%	6	19.35%			
**Type Activities**	**fc**	**(%)**	**fc**	**(%)**	**χ^2^**	***p***	***V***
Individual sport	3	10.34%	1	3.70%	6.937	0.139	0.352
Collective sport	11	37.93%	11	40.74%			
Fitness/muscle building	7	24.14%	5	18.52%			
Fitness/muscle building/sport	3	10.34%	9	33.33%			
Individual and collective sport	5	17.24%	1	3.70%			

fc = frequency; χ2 = chi-squared; *p* = significance; *V* = Cramér’s *V*, *V* < 0.10 = irrelevant effect, if 0.10 < *V* < 0.30 = small effect, if 0.30 < *V* < 0.50 = moderate effect, if *V* > 0.50 effect big.

**Table 4:** Evaluation of the intervention: changes in sports habits.

Weekly Frequency							
**Control Group**	*Post-intervention*				***M***	***p***	***V***
*Pre-intervention*	Between 1 and 2 days	Between 3 and 4 days	5 or more days	Total	2.700	0.440	0.204
Between 1 and 2 days	4	3	0	7			
Between 3 and 4 days	5	11	2	18			
5 or more days	2	3	1	6			
Total	11	17	3				
**Experimental Group**	*Post-intervention*				***M***	***p***	***V***
*Pre-intervention*	Between 1 and 2 days	Between 3 and 4 days	5 or more days	Total	7.500	0.058	0.349
Between 1 and 2 days	6	7	1	14			
Between 3 and 4 days	1	7	2	10			
5 or more days	5	1	1	7			
Total	12	15	4				
**Duration of the Session**							
**Control Group**	*Post-intervention*				***M***	***p***	***V***
*Pre-intervention*	Between 30 and 60 min	Between 30 and 60 min	More than 90 min	Total	4.143	0.126	0.398
Between 30 and 60 min	4	4	0	8			
Between 30 and 60 min	3	13	0	16			
More than 90 min	0	4	1	5			
Total	7	32	2				
**Experimental Group**	*Post-intervention*				***M***	***p***	***V***
*Pre-intervention*	Between 1 and 2 days	Between 3 and 4 days	5 or more days	Total	4.143	0.126	0.319
Between 1 and 2 days	1	3	0	4			
Between 3 and 4 days	4	17	0	21			
5 or more days	0	4	1	5			
Total	5	24	1				

*M* = McNemar–Bowker test; *p* = significance; *V* = Cramér’s *V*, *V* < 0.10 = irrelevant effect, if 0.10 < *V* < 0.30 = small effect, if 0.30 < *V* < 0.50 = moderate effect, if *V* > 0.50 effect big.

**Table 5:** Evaluation of the intervention: changes in the appreciation of customer satisfaction.

		Pre-Intervention		Post-Intervention		Significance			Evolution	
		Control Group	Exp. Group	Control Group	Exp. Group	Main Effects		Interaction	Control Group	Exp. Group
						Group	Time			
satisfaction v1	N	31	29	31	29	F = 0.113	F = 8.253	F = 0.146		
	M	4.323	4.241	4.613	4.621	*p* = 0.738	*p* = 0.006 *	*p* = 0.704	Mdif = −0.290	Mdif = −0.379
	SD	0.653	0.689	0.558	0.561	η^2^_p_ = 0.002	η^2^_p_ = 0.125	η^2^_p_ = 0.003	t = 1.792	t = 2.264
									*p* = 0.288	*p* = 0.119
satisfaction v2	N	30	30	30	30	F = 4.204	F = 8.646	F = 0.001		
	M	4.333	4.100	4.733	4.500	*p* = 0.045 *	*p* = 0.005 *	*p* = 1.000	Mdif = −0.400	Mdif = −0.4
	SD	0.884	0.712	0.450	0.630	η^2^_p_ = 0.068	η^2^_p_ = 0.130	η^2^_p_ = 0.001	t = 2.079	t = 2.079
									*p* = 0.172	*p* = 0.172
satisfaction v3	N	29	30	29	30	F = 0.098	F = 18.496	F = 2.867		
	M	4.069	4.300	4.759	4.600	*p* = 0.755	*p* < 0.001 *	*p* = 0.096	Mdif = −0.690	Mdif = −0.300
	SD	0.651	0.794	0.511	0.498	η^2^_p_ = 0.002	η^2^_p_ = 0.245	η^2^_p_ = 0.048	t = 4.203	t = 1.860
									*p* < 0.001 *	*p* = 0.257
satisfaction v4	N	29	31	29	31	F = 0.241	F = 13.157	F = 0.307		
	M	4.172	4.290	4.655	4.645	*p* = 0.625	*p* < 0.001 *	*p* = 0.582	Mdif = −0.483	Mdif = −0.355
	SD	0.759	0.588	0.484	0.608	η^2^_p_ = 0.004	η^2^_p_ = 0.185	η^2^_p_ = 0.005	t = 2.909	t = 2.210
									*p* = 0.026 *	*p* = 0.133
satisfaction v5	N	29	31	29	31	F = 2.022	F = 10.975	F = 0.197		
	M	4.345	4.129	4.690	4.581	*p* = 0.160	*p* = 0.002 *	*p* = 0.659	Mdif = −0.345	Mdif = −0.452
	SD	0.614	0.763	0.471	0.672	η^2^_p_ = 0.034	η^2^_p_ = 0.159	η^2^_p_ = 0.003	t = 1.995	t = 2.702
									*p* = 0.202	*p* = 0.044 *

* Denotes statistically significant differences; N = sample size; M = mean; SD = standard deviation; F = F test; *p* = significance; η^2^_p_ = partial eta squared; if η^2^_p_ < 0.01 trivial effect, if 0.01 < η^2^_p_ <0.06 small effect, if 0.06 < η^2^_p_ < 0.14 medium effect, if η^2^_p_ > 0.14 important effect. V1: In general, I am satisfied with the staff of the fitness center; v2: In general, I am satisfied with the facilities and the material. v3: In general, I am satisfied with the services and activities that are offered. v4: I am satisfied with the quality/price ratio of the service in general. v5: My impression of the organization in general is good.

**Table 6:** Evaluation of the intervention: changes in the maintenance intention in the fitness center.

		Pre-intervention		Post-intervention		Significance			Evolution	
		Control Group	Exp. Group	Control Group	Exp. Group	Main Effects		Interaction	Control Group	Exp. Group
						Group	Time			
intention v1	N	30	30	30	30	F = 1.396	F = 3.120	F = 0.005		
	M	4.633	4.467	4.400	4.233	*p* = 0.242	*p* = 0.083	*p* = 1.000	Mdif = 0.233	Mdif = 0.233
	SD	0.809	0.681	0.724	0.774	η^2^_p_ = 0.023	η^2^_p_ = 0.051	η^2^_p_ = 0.001	t = 1.249	t = 1.249
									*p* = 0.599	*p* = 0.599
intentionv2	N	28	31	28	31	F = 0.158	F = 14.716	F = 0.166		
	M	4.000	4.000	4.679	4.548	*p* = 0.692	*p* < 0.001 *	*p* = 0.686	Mdif = −0.679	Mdif = −0.548
	SD	1.054	1.125	0.548	0.624	η^2^_p_ = 0.003	η^2^_p_ = 0.205	η^2^_p_ = 0.003	t = 2.927	t = 2.489
									*p* = 0.025 *	*p* = 0.072
intention v3	N	30	30	30	30	F = 2.707	F = 20.625	F = 0.098		
	M	3.600	3.300	4.633	4.200	*p* = 0.105	*p*<0.001 *	*p* = 0.755	Mdif = −1.033	Mdif = −0.900
	SD	1.354	1.512	0.615	1.095	η^2^_p_ = 0.045	η^2^_p_ = 0.262	η^2^_p_ = 0.002	t = 3.433	t = 2.990
									*p* = 0.006*	*p* = 0.021*
intention v4	N	29	30	29	30	F = 1.285	F = 1.516	F = 0.197		
	M	3.621	3.800	3.793	4.167	*p* = 0.262	*p* = 0.223	*p* = 0.659	Mdif = −0.172	Mdif = −0.367
	SD	1.237	1.297	1.207	1.289	η^2^_p_ = 0.022	η^2^_p_ = 0.026	η^2^_p_ = 0.003	t = 0.552	t = 1.195
									*p* = 0.947	*p* = 0.633
intention v5	N	31	28	31	28	F = 4.996	F = 9.426	F = 0.081		
	M	3.323	3.964	3.968	4.500	*p* = 0.029 *	*p* = 0.003	*p* = 0.777	Mdif = −0.645	Mdif = −0.536
	SD	1.326	1.374	1.303	0.923	η^2^_p_ = 0.081	η^2^_p_ = 0.142	η^2^_p_ = 0.001	t = 2.435	t = 1.921
									*p* = 0.082	*p* = 0.231
intention v6	N	29	31	29	31	F = 1.511	F = 16.595	F = 3.533		
	M	3.138	3.806	4.276	4.226	*p* = 0.224	*p* < 0.001 *	*p* = 0.065	Mdif = −1.138	Mdif = −0.419
	SD	1.529	1.223	0.922	1.146	η^2^_p_ = 0.025	η^2^_p_ = 0.222	η^2^_p_ = 0.057	t = 4.141	t = 1.578
									*p* < 0.001 *	*p* = 0.399
intention v7	N	28	31	28	31	F = 0.964	F = 19.850	F = 1.397		
	M	3.500	3.452	4.500	4.032	*p* = 0.330	*p* < 0.001 *	*p* = 0.242	Mdif = −1.000	Mdif = −0.581
	SD	1.374	1.434	0.882	1.080	η^2^_p_ = 0.017	η^2^_p_ = 0.258	η^2^_p_ = 0.024	t = 3.889	t = 2.376
									*p* = 0.001 *	*p* = 0.093

* Denotes statistically significant differences; N = sample size; M = mean; SD = standard deviation; F = F test; *p* = significance; η^2^_p_ = partial eta squared; if η^2^_p_ < 0.01 trivial effect. if 0.01 < η^2^_p_ < 0.06 small effect, if 0.06 < η^2^_p_ < 0.14 medium effect, if η^2^_p_ > 0.14 important effect. V1: best price, v2: closer to the workplace, v3: closer to home, v4: better equipment, v5: better facilities, v6: better technical equipment, v7: better technological equipment.

## Data Availability

The data presented in this study are available on request from the corresponding authors.
